# Causal associations between insulin-like growth factor binding protein-1 to -7 and osteoporosis: A two-sample Mendelian randomization study

**DOI:** 10.1097/MD.0000000000045227

**Published:** 2025-11-21

**Authors:** Peng Du, Tusheng Li, Ning Fan, Baodong Wang, Tianyi Wang, Lei Zang

**Affiliations:** a Department of Orthopedics, Beijing Chaoyang Hospital, Capital Medical University, Beijing, China.

**Keywords:** bone metabolism, causal inference, insulin-like growth factor binding protein, Mendelian randomization, osteoporosis

## Abstract

Insulin-like growth factor binding proteins (IGFBPs) are important regulatory factors of bone metabolism. However, the association between IGFBPs and osteoporosis remains a subject of contention in observational studies. We conducted a 2-sample Mendelian randomization (MR) study to assess the causal relationship between IGFBP 1-7 and osteoporosis using publicly available genome-wide association study summary statistics. The IGFBP 1-7 datasets were derived from German cohort studies and were selected at the genome-wide significance level (*P *< 5 × 10^-6^). The osteoporosis dataset was obtained from the UK Biobank study and included 484,598 individuals of European ancestry. Inverse variance weighting (IVW) was used as the primary MR method to investigate the causal relationship between exposure and outcome. To make the conclusions more robust and reliable, we used Cochran Q statistics, MR-Egger regression, and leave-one-out analysis as sensitivity analyses. Based on the IVW results, we identified a causal relationship between IGFBP-2 and osteoporosis, in which IGFBP-2 prevented the development of osteoporosis (IVW, *P *= .006; odds ratio = 0.998; β = −0.0022; 95% confidence interval: 0.996–0.999). However, the study did not show a significant causal relationship between the remaining 6 IGFBPs and osteoporosis (*P *> .05). In addition, the sensitivity analyses confirmed the findings in our study were robust. This MR study suggests a potential protective role of IGFBP-2 against osteoporosis and that it may serve as a biomarker or therapeutic target, although further research is needed.

## 1. Introduction

Osteoporosis is a common systemic skeletal disorder that results from multiple factors and is characterized by a decrease in bone density, degeneration of bone microstructure, and an increased susceptibility to fractures.^[1,2]^ A global epidemiological study on osteoporotic vertebral fractures reported that more than 200 million people worldwide are affected by osteoporosis.^[3]^ It is estimated that the number of people with osteoporosis in the United States will reach 121.3 million in 2025, imposing an annual burden on healthcare resources exceeding $25.3 billion.^[4]^ Clearly, the serious clinical and economic consequences of osteoporosis demand urgent investigation so that more effective treatments can be developed. Although current clinical interventions for osteoporosis, such as administration of calcitonin, bisphosphonates, or estrogen receptor modulators effectively restore bone strength, they are associated with significant side effects, including fever, hypercalcemia, bone pain, endometrial cancer, breast cancer, and cardiovascular diseases.^[5,6]^ Therefore, the development of novel and effective therapeutic strategies is of paramount importance and requires comprehensive research to be undertaken to determine the factors involved in the pathogenesis of osteoporosis.

The insulin-like growth factor (IGF) system is a crucial regulatory component of human bone metabolism and consists of IGFs (IGF-1 and IGF-2), IGF receptors, and IGF binding proteins (IGFBPs).^[7]^ IGFs are the most abundant growth factors produced by osteoblasts and have a protective role in osteoporosis by enhancing bone formation through the regulation of osteoblast proliferation, differentiation, and apoptosis.^[8,9]^ Previous studies have shown that the IGFBP family has a huge impact on the biological activity of IGFs, which can be modulated by inhibiting the binding of IGF to their receptors and/or regulating the amount of IGFs in the circulation.^[7,10]^ Given the limitations of systemic administration of IGFs, the current focus is on using IGFBPs to modulate local IGFs levels in bone tissue.^[11]^ Circulating IGFBPs consist primarily of 6 high-affinity IGFBPs (IGFBP 1-6) and one low-affinity IGFBP (IGFBP-7), which regulate the effects of IGFs on bone metabolism in either a positive or negative manner. Currently, some researchers consider that IGFBP-1, 2, 4, and 6 inhibit osteoblast function to reduce bone formation, IGFBP-3 and 5 stimulate osteoblast function to increase bone formation, while IGFBP-7 restrains osteoclast activity to attenuate osteoporotic bone loss.^[6,8–10]^ However, an increasing body of evidence suggests that IGFBPs simultaneously enhance and inhibit the actions of IGFs, dependent largely on cell phenotype, the relative concentrations of IGFBPs and ligands, post-translational modifications, and the non-receptor-mediated effects of IGFs.^[9,11,12]^ Therefore, the exact role of IGFBPs in bone metabolism remains incompletely understood, and as a result, their association with osteoporosis is a subject of ongoing debate in current research.

Mendelian randomization (MR) is an effective method that uses genetic variation as an instrumental variable (IV) to study causal relationships between exposure and outcome phenotypes.^[13]^ MR can effectively addresses the issues of confounding and reverse causality effects present in observational studies. To ascertain whether a causal relationship exists between IGF-BPs and osteoporosis, we conducted a 2-sample MR analysis using publicly accessible genome-wide association study (GWAS) datasets. A GWAS involves scanning markers across the complete sets of DNA, or genomes, of many people to find genetic variations associated with a particular disease. Once new genetic associations they can be used to develop better strategies to detect, treat and prevent the disease. To the best of our knowledge, no MR study has investigated the relationship between IGFBP 1-7 and osteoporosis, while no randomized controlled trial (RCT) has extensively investigated their causal associations.

## 2. Materials and methods

### 2.1. Study design

Three critical assumptions must be satisfied in MR studies to obtain reliable and robust results.^[14]^ The first assumption requires that the genetic variables are associated significantly with the exposure of interest. The second assumption demands that the genetic variables used as IVs for the exposure are independent of other confounding factors, while the third assumption asserts that genetic variables can only affect the outcome through their impact on exposure, thereby precluding horizontal pleiotropic effects. Based on publicly available GWAS summary statistics and the results from recent observational studies, we initially selected 7 distinct IGFBPs as exposure factors. The IGFBP 1-7 datasets were sourced from a German cohort study. We then carefully selected IVs for each subtype of IGFBP to identify the largest cohort study on osteoporosis within the GWAS summary statistics as our outcome dataset. The osteoporosis dataset was obtained from the UK Biobank website (https://www.ukbiobank.ac.uk/) and included 484,598 individuals of European ancestry. All these research studies have received approval from the relevant Institutional Review Boards, obviating the need for additional Institutional Review Board approval.

**Figure 1.**
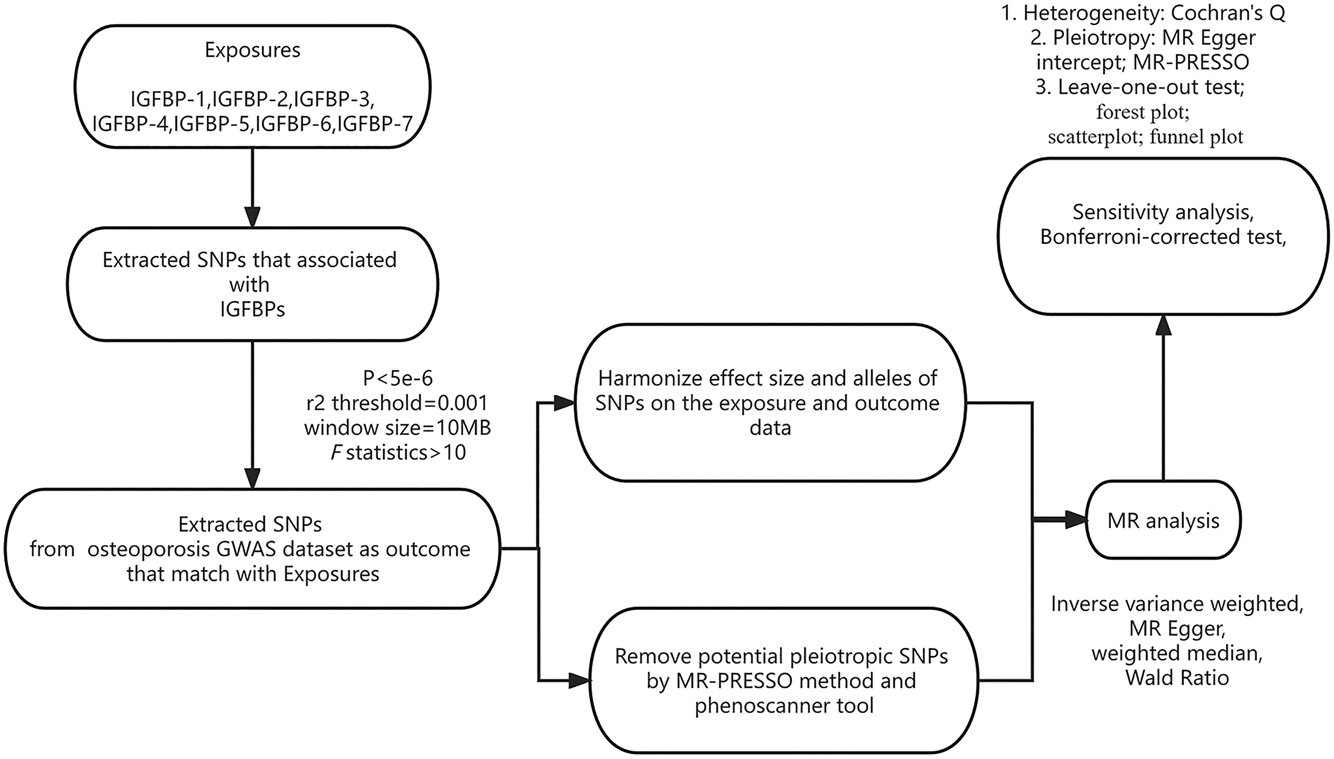
Flowchart of Mendelian randomization study design. Analyses were performed using the TwoSampleMR version 0.5.7 package in the R software. All data are derived from publicly available GWAS summary statistics. GWAS = genome-wide association study, IGFBP = insulin-like growth factor binding protein, MR = Mendelian randomization, SNP = single-nucleotide polymorphism.

A 2-sample MR analysis was conducted to estimate the causal effects between different forms of IGFBPs and osteoporosis. All the analyses were performed using the 2 samples MR package (version 0.5.6) in R software (version 4.3.1). The threshold of a *P* value that indicated statistical significance in the MR study was set below .007 (0.05/7 by Bonferroni correction). Figure 1 illustrates the overall design of the study, while Table 1 provides an overview of the data sources used in this research.

**Table 1:** Phenotypes analyzed and GWAS summary statistics selected as exposure/outcome data.

Phenotype	GWAS ID	Sample size	SNPs	Year
IGFBP-1	prot-a-1452	3301	10534735	2018
IGFBP-2	prot-a-1448	3301	10534735	2018
IGFBP-3	prot-a-1449	3301	10534735	2018
IGFBP-4	prot-c-2950_57_2	NA	501428	2019
IGFBP-5	prot-c-2685_21_2	NA	501428	2019
IGFBP-6	prot-a-1450	3301	10534735	2018
IGFBP-7	prot-c-3320_49_2	NA	501428	2019
Osteoporosis	ebi-a-GCST90038656	484598	9587836	2021

NA: Data from PMID: 28240269, no explicit sample size information reported in the literature or GWAS summary data.

### 2.2. Data sources

All the data used in the study were sourced from summary statistics provided by the GWAS platform (https://gwas.mrcieu.ac.uk/). To the best of our knowledge, this represents the largest sample size for a GWAS study to date that was consistent with our study design. The GWAS summary statistics for IGFBP1-7 and osteoporosis were downloaded from the publicly available GWAS catalogue website (https://www.ebi.ac.uk/gwas/downloads/summary-statistics). Their IDs are shown in Table 1.

### 2.3. Statistical methods

The study used various MR methods in conjunction with summary statistics to determine the causal relationship between each of the 7 types of IGFBPs and osteoporosis. Based on different assumptions, 3 MR methods were used to validate the results obtained. These included inverse variance weighting (IVW),^[15]^ weighted mean,^[16]^ and MR-Egger regression.^[17]^ The IVW method served as the primary statistical model. However, it is important to note that IVW is susceptible to bias or multiple effects due to the influence of invalid IVs. To ensure the validity and robustness of our findings, we conducted a series of sensitivity analyses. Both the fixed-effects and the random-effects IVW methods were used for our analysis. Initially, we calculated causal estimates using the fixed-effects IVW method, while for cases where significant heterogeneity (*P* < .05) was observed, we incorporated the random-effects IVW method. If only one IV was screened, the Wald ratio method was used. In addition, the F statistic for each IV was calculated, with an- *F* > 10 indicating the absence of IV bias.^[18]^

### 2.4. Sensitivity analysis

The interpretation of causal estimates derived from the MR study was dependent on satisfying 3 critical assumptions. Heterogeneity in causality estimates between IVs may indicate potential violations of these MR study assumptions. Cochran Q test was used to investigate the heterogeneity in the causal estimates, utilizing both the causal estimates obtained from the fixed-effects IVW method and MR-Egger regression. The degree of heterogeneity was quantified using Cochran Q statistics, with statistical significance set at *P* < .05 to indicate substantial heterogeneity. In addition, we used the MR-Egger regression method to assess the potential existence of pleiotropic effects associated with the IVs. This approach determined whether or not the IVs exhibited directional pleiotropy and also provided an estimate of the causal effect. The presence of horizontal pleiotropy in the causal estimates was shown as the intercept term in the MR-Egger regression.^[19]^ In cases where horizontal pleiotropy was detected among the IVs, PhenoScanner software (https://www.ebi.ac.uk/fg/phenoscanner/) was used to identify and exclude any IVs that might have influenced the outcome.^[20]^ In addition to the primary analyses, a leave-one-out analysis was conducted which systematically in a stepwise manner excluded each single-nucleotide polymorphism (SNP). We then performed the MR study on the remaining SNPs to identify potential outlier IVs.

## 3. Results

### 3.1. Instrumental variables

The SNPs associated with the 7 IGFBPs at a genome-wide significance level (*P* < 5E − 6) were used as the candidate IVs for our subsequent MR study. To ensure the independence of the IVs for each exposure phenotype, linkage disequilibrium (LD)-based clumping was used to remove SNPs with a strong LD (*r*^2^ = 0.001, window size = 10 MB). This clumping process relied on the European reference panel of the 1000 Genomes Project to estimate the LD between SNPs. For SNPs that were not present in the exposure GWAS data, we identified proxy SNPs that showed the strongest correlation with data from the 1000 Genomes European population (*r*^2^ > 0.8). The SNPs with non-concordant alleles and palindromic SNPs with ambiguous strands which could not be resolved during harmonization of the exposure and outcome data were also excluded. These rigorously filtered SNPs were subsequently employed as IVs for our MR study. A SNP rs9855615 was removed from the MR study of IGFBP-3 for being palindromic with intermediate allele frequencies. In the study, all the *F* statistics were >10, indicating a low likelihood of weak IV bias. In addition, MR-Egger regression showed that there was no horizontal pleiotropy (*P *> .05) in all the screened SNPs. The results of the sensitivity analyses are summarized in the Supplementary Materials (Supplementary Image 1, Supplemental Digital Content, https://links.lww.com/MD/Q530).

### 3.2. MR study of the causal relationship between IGFBP 1-7 and osteoporosis

**Figure 2.**
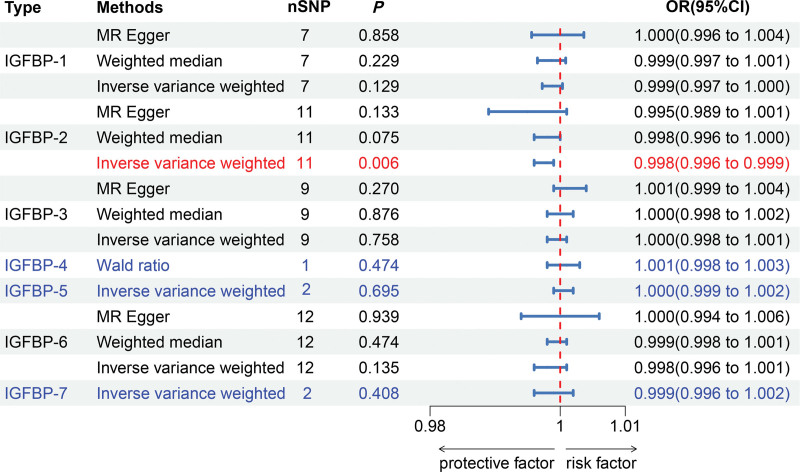
MR study of the effect of IGF-BP 1-7 levels on the risk of osteoporosis. The IVW results served as the primary findings in the MR analysis, while the remaining methods functioned as points of reference. If the odds ratio (OR) values from the other methods exhibited consistent directional alignment with those from the IVW method, it could be inferred that the other methods also corroborated the IVW results. Statistical significance was determined at a *P*-value threshold of < 0.007. Consequently, the analysis revealed a causal correlation only between IGF-BP2 and osteoporosis. For MR analysis employing a single SNP as the instrumental variable, the Wald ratio was utilized. When 2 SNPs were employed as instrumental variables, the IVW method exclusively guided the analysis. GWAS = genome-wide association study, IGFBP = insulin-like growth factor binding protein, IVW = inverse variance weighting, MR = Mendelian randomization, SNP = single-nucleotide polymorphism.

The dataset with the largest sample size of IGFBP1-7 from the GWAS summary statistics was used as the exposure data, while the dataset with the largest sample size of osteoporosis was used as the outcome data. The results of the MR study are shown in Figure 2 and Table 2. Based on the IVW obtained, we observed a negative causal relationship between IGFBP-2 and osteoporosis (IVW: *P *= .006; odds ratio [OR] = 0.998; β = −0.0022; 95% confidence interval [CI]: 0.996–0.999). This suggested that IGFBP-2 had a preventive effect on the development of osteoporosis. It is important to note that an increase of 100 standard deviations in IGFBP-2 levels decreased the risk of developing osteoporosis by 20%. In addition, this causal relationship between IGFBP-2 and osteoporosis remained in initial practice using the adjusted *P*-values (*P *< .007) after Bonferroni correction, which reinforced the validity of our conclusions. However, the present study did not show a significant causal relationship between the remaining 6 IGFBPs and osteoporosis (*P *> .05), including IGFBP-1 (IVW, *P *= .129; OR = 0.999; β = −0.0012; 95% CI, 0.997–1.000), IGFBP-3 (IVW, *P *= .758; OR = 1.000; β = −0.0002; 95% CI, 0.998–1.001), IGFBP-4 (WR, *P *= .474; OR = 1.001; β = 0.0009; 95% CI, 0.998–1.003), IGFBP-5 (IVW, *P *= .695; OR = 1.000; β = 0.0004; 95% CI, 0.999–1.002), IGFBP-6 (IVW, *P *= .135; OR = 0.998; β = −0.0017; 95% CI, 0.996–1.001), IGFBP-7 (IVW, *P *= .408; OR = 0.999; β = −0.0011; 95% CI, 0.996–1.002).

**Table 2:** Effect values of MR studies and results of heterogeneity test and pleiotropy test.

Exposures	Method	β	SE	Q-pval	Intercept	M-pval
IGFBP-1	IVW	−0.0012	0.0008	0.124	0.0005	0.681
IGFBP-2	IVW	−0.0022	0.0008	0.599	−0.0001	0.367
IGFBP-3	IVW	−0.0002	0.0008	0.157	−0.0004	0.135
IGFBP-4	Wald ratio	0.0009	0.0013	/	/	/
IGFBP-5	IVW	0.0004	0.0009	0.641	/	/
IGFBP-6	IVW	−0.0017	0.0011	<0.001	−0.0003	0.494
IGFBP-7	IVW	−0.0011	0.0014	0.017	/	/

Method: we describe the primary method employed in Mendelian randomization and provide the corresponding β-values and standard errors (SE) generated by the method.

IVW = inverse variance weighted, Q-pval = This represents the *P*-value of the heterogeneity test using Cochran Q statistics. A value <0.05 indicates the presence of heterogeneity in the IVs, leading to the use of the IVW method with a random-effects model. If the *P*-value is >.05, the fixed-effects model is employed; Intercept: This denotes the intercept term in MR-Egger regression. An intercept close to 0 indicates the absence of horizontal pleiotropy in the IVs; M-pval: It signifies the significance of horizontal pleiotropy assessed using the MR-PRESSO method. A value >0.05 suggests the absence of horizontal pleiotropy. “/”: This symbol indicates that a particular method is not applicable due to an insufficient number of IVs

As shown in Table 2, the SNPs for IGFBP-6 and IGFBP-7 exhibited heterogeneity, leading us to adopt the IVW approach within a random-effects model to ensure our results were robust. Furthermore, it is worth noting that despite the limited number of IVs available for IGFBP-4, IGFBR-5, and IGFBP-7, our selection of IVs for each of these binding proteins as exposure factors was screened rigorously. Therefore, even if only a single IV was associated with the results, it remained informative. No horizontal pleiotropy was detected in the IVs for any of the 7 IGFBPs, as indicated by the intercepts closely approximating 0. The funnel plots, scatter plots, and forest plots for the MR study are available in the Supplementary Material (Supplementary Image 11, Supplemental Digital Content, https://links.lww.com/MD/Q530), with the findings showing a high degree of robustness.

## 4. Discussion

This study used GWAS summary statistics to analyze the causal relationship between IGFBP 1-7 and osteoporosis. The results indicate a negative correlation between IGFBP-2 and the development of osteoporosis, suggesting that higher concentrations of IGFBP-2 may be associated with a reduced risk of osteoporosis. Notably, although the effect size (β = −0.0022) between IGFBP-2 and osteoporosis seems not large, it indicates that an increase of 100 standard deviations in IGFBP-2 levels decreased the risk of developing osteoporosis by 20%. Importantly, this causal relationship between IGFBP-2 and osteoporosis remained in initial practice using the adjusted *P*-values (*P* < .007) after Bonferroni correction, which reinforced the validity of our conclusions. However, there was no evidence to support a causal relationship between osteoporosis and IGFBP-1 and IGFBP 3–7. To the best of our knowledge, this is the first 2-sample MR study that investigated the causal relationship between IGFBPs and osteoporosis. The study successfully demonstrated a negative causal link between IGFBP-2 and osteoporosis, evidence which may contribute to prevention and early intervention in individuals with a high risk of developing osteoporosis.

The role of IGFBPs in bone formation and resorption has long been a focus of research, with the results indicating that they may serve as effective biological marker targets for the prevention and treatment of osteoporosis. Nevertheless, there is considerable controversy in current observational studies regarding the impact of IGFBPs on bone metabolism, necessitating further research to provide stronger evidence. IGFBP-2 has been a particularly contentious subject among the IGFBPs, due mainly to observational studies reporting inconsistent results. Although some scholars consider that IGFBP-2 acts as a negative regulator of IGFs in bone formation by inhibiting osteoblast proliferation and differentiation, and collagen synthesis,^[8–10]^ there is evidence suggesting that IGFBP-2 promotes skeletal metabolism and prevents bone loss.^[11,21,22]^ In this regard, the present study showed a negative correlation between IGFBP-2 and osteoporosis, indicating that it has a preventive effect on the development of osteoporosis, a finding consistent with previous observational studies. For example, Khosla and colleagues^[23,24]^ reported that IGFBP-2 stimulated bone formation in patients with hepatitis C-related osteosclerosis, with this change associated with an increase in IGF-2, while an in vitro study in rats by Palermo et al^[25]^ showed that the addition of equimolar concentrations of IGFBP-2 had a synergistic effect on IGF-2-mediated differentiation in osteoblast cells. Another rat study also showed that a peptide containing the IGFBP2 receptor binding site enhanced bone mass in ovariectomized rats, leading the authors to conclude that IGFBP2 was necessary for maintaining bone mass in the absence of estrogen.^[21]^ Furthermore, Zhou et al^[22]^ discovered that IGF2BP-2 was a RNA-binding protein that stabilized the serum response factor mRNA to regulate cell proliferation and osteogenic differentiation, suggesting that IGF2BP-2 was a potential therapeutic target for treating osteoporosis. Circulating IGFBP-2 binds to IGFs with high affinity and modulates the effects that IGFs have on bone metabolism by regulating the amount of IGF-1 and IGF-2 transported out of the vasculature.^[26]^ IGF-2 is the predominant IGF present in the human skeleton and is produced by osteoblasts at a rate several times faster than IGF-1.^[27]^ In addition, IGFBP-2 is the only IGFBP that enhances the promotion of matrix binding induced by IGF-2, with the IGF-2/IGFBP-2 complex preferentially targeting bone to promote in vivo bone anabolism by increasing bone mineral density.^[11]^ This suggests that IGF-2/IGFBP-2 complexes may have superior effects on bone cells, although further research is needed to confirm this association.

Based on the findings of these previous studies, the main mechanisms by which IGFBP-2 promotes bone formation are as follows: IGFBP-2 promotes the proliferation and differentiation of osteoblasts by increasing the concentration of IGFs. Some studies have indicated that IGFBP-2 has a strong tendency to bind with extracellular matrix components, thereby increasing the local concentration of IGF-1 available for receptor binding and enhancement of bone formation.^[28]^ IGFBP-2 may act in a manner that is not completely dependent on IGFs, with studies showing that it stimulates osteoblast differentiation and bone formation by binding directly to receptor tyrosine phosphatase beta (RPTPβ).^[21]^ The GFBP2/RPTPβ signaling pathway acts in coordination with the IGF-1 receptor-associated signaling pathway. Binding of IGFBP-2 to RPTPβ not only decreases the activity of protein kinase B (AKT) inhibitors and the phosphatase and tensin homolog (PTEN), but enhances the ability of IGF-1 to stimulate AKT activation, thereby enhancing IGFBP-2 stimulation of bone conversion.^[21,28–30]^ IGFBP-2 is a crucial important regulator for increasing vitamin D concentration and decreasing liver fat levels in vivo.^[31,32]^ Studies have demonstrated that high concentrations of vitamin D and low levels of liver fat are important factors in promoting bone growth and/or inhibiting bone destruction,^[33,34]^ which may be another potential mechanism by which IGFBP2 prevents osteoporosis.

IGFBPs exert varying effects on osteoporosis by enhancing and/or inhibiting the impact of IGFs on bone formation. Previous research has shown that aside from IGFBP-2, IGFBP-3, -4, and -5 also exhibit a dual role that includes both positive and negative aspects under the influence of various factors.^[8,9]^ IGFBP-1 and -6 are generally considered to have inhibitory effects, while IGFBP-7 prevents osteoporosis by reducing bone loss.^[6,10]^ However, the current study showed no significant causal relationship between osteoporosis and IGFBP-1 or IGFBP-3 to -7, a finding which differs somewhat from many published observational studies. The reasons for the differences between the results of our MR analysis and those of other observational studies can be explained as follows. First, the results from observational studies are often influenced by other related factors. For example, IGFBP-3 is regarded widely as a positive regulator of bone metabolism, thereby offering protection against osteoporosis. A prospective double-blind trial by Boonen et al^[35]^ reported that administering a complex of IGF-1 coupled with IGFBP-3 reduced femoral bone loss and improved functional outcomes in elderly patients with a hip fracture. However, in a cross-sectional study of 85 postmenopausal women, Shi et al^[36]^ examined the relationship between IGFBP-3 and bone mineral density at the lumbar spine and femoral neck and found that IGFBP-3 levels were significantly higher in the osteoporosis group than in the normal group, with IGFBP-3 inversely correlated with bone mineral density. Secondly, the results of our MR analysis may have been affected by bias due to pleiotropy. This is an inherent limitation of MR studies, although in the current study we mitigated this issue by screening genetic variants associated with confounding factors using PhenoScanner data and conducting MR-PRESSO outlier tests to identify and remove outlier variants. Therefore, we can reasonably consider that the possibility of pleiotropy significantly influencing the results of our analysis is low. In addition, we performed various robust MR methods such as IVW, weighted median, and the MR-Egger test, which made our conclusions stronger and more reliable.

This study had several strengths and limitations. The strengths lie primarily in the design of the MR study, which effectively reduced bias from confounding factors and reversed causality that can be present in observational studies.^[37]^ The study was also based on large-scale datasets that were used to analyze the causal relationship between IGFBP 1-7 and osteoporosis that significantly enhanced the persuasiveness of our conclusions. Moreover, the exposure and outcome datasets were derived from different populations, thereby eliminating issues related to sample overlap. The limitations of our study, included that it was only in enrolled individuals of European ancestry, which may have constrained the generalizability of our findings to other populations. We also cannot completely rule out the possibility that pleiotropy may have influenced our results, even though we took several measures to screen genetic variants associated with confounding factors and systematically removed outliers. Furthermore, although MR shares similarities with RCTs in addressing confounding factors and reverse causality issues in observational studies, MR cannot provide the same level of causal inference as RCT studies. High-quality RCT studies are still needed in the future to clarify the relationship between IGFBP-2 and osteoporosis.

## 5. Conclusion

In summary, this MR study suggests a potential protective role of IGFBP-2 against osteoporosis and that it may serve as a biomarker or therapeutic target, although further research is needed. However, the study did not find a causal relationship between osteoporosis and IGFBP-1 or IGFBP 3–7. Therefore, in the future, when more advanced methods are available to reduce biased estimates, improve precision, and obtain GWAS pooled data with larger sample sizes, it will be necessary to perform updated MR analyses to confirm our findings.

## Acknowledgments

The authors are grateful for all the coalition research that has made the aggregated association statistics publicly available, and for the expert linguistic services provided by EditSprings (https://www.editsprings.cn).

## Author contributions

**Conceptualization:** Lei Zang, Ning Fan, Baodong Wang.

**Data curation:** Ning Fan, Baodong Wang, Tianyi Wang.

**Formal analysis:** Peng Du, Tusheng Li, Ning Fan, Baodong Wang.

**Writing – original draft:** Peng Du, Tusheng Li.

**Writing – review & editing:** Lei Zang, Peng Du, Tusheng Li, Tianyi Wang.

## Supplementary Material


