# Supplementary material for: Causal associations between insulin-like growth factor binding protein-1 to -7 and osteoporosis: A two-sample Mendelian randomization study
Source: Medicine (Baltimore). 2025 Nov 21;104(47):e45227. doi: 10.1097/MD.0000000000045227 (PMC12643744; doi:10.1097/MD.0000000000045227)
Supplement: Supplementary file 1 [file medi-104-e45227-s001.docx]

**
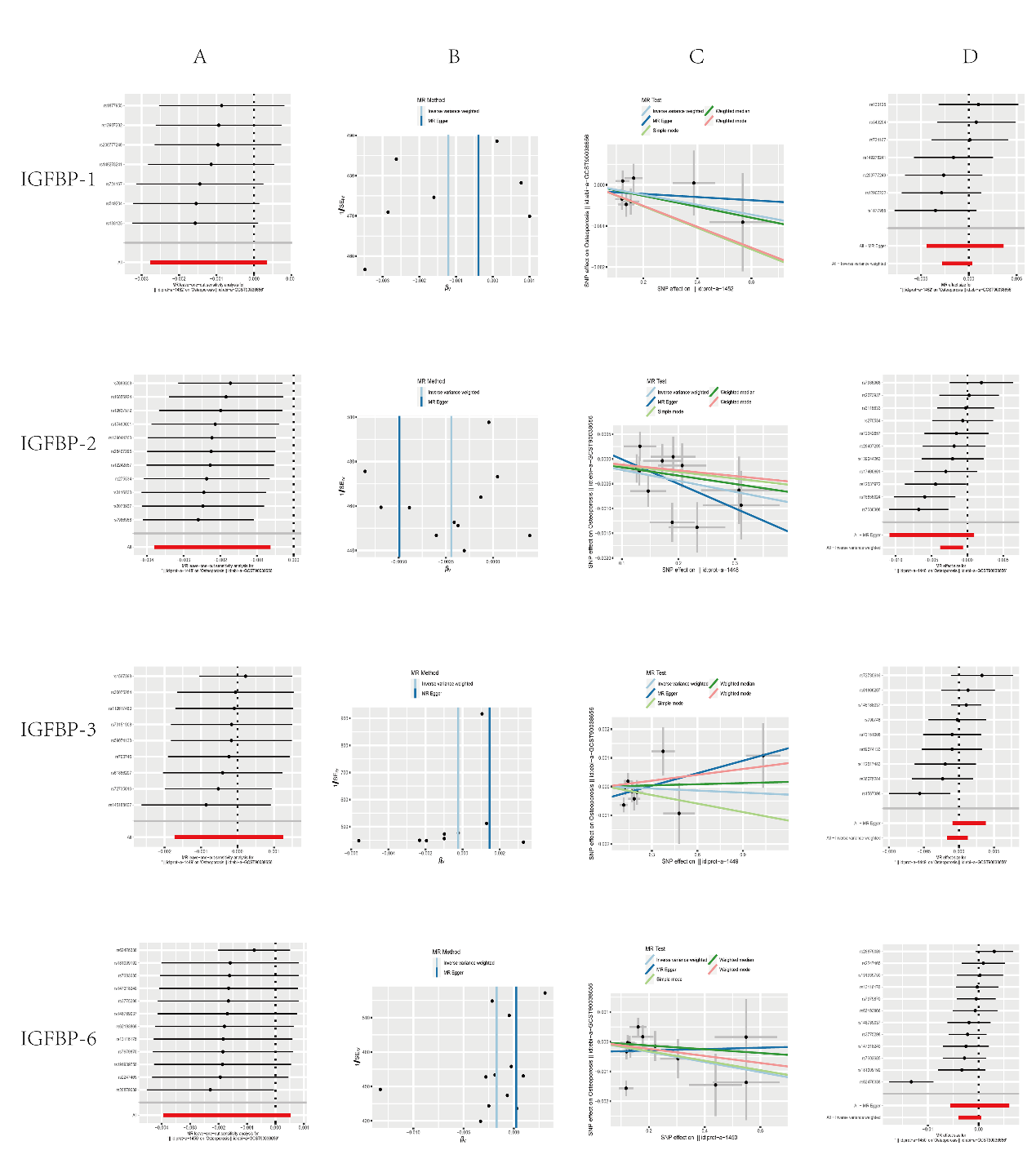
**

**Supplementary Figure 1**: Summary of Mendelian Randomization (MR) Results

A: Leave-One-Out Analysis

The leave-one-out method involves a step-by-step exclusion test, where each SNP is removed one by one. The meta-effect of the remaining SNPs is calculated. If the overall error does not change significantly after each SNP removal, the results are considered reliable.

B: Funnel Plot for Heterogeneity

The funnel plot assesses the heterogeneity of SNPs, focusing on the symmetry of points on the left and right sides of the IVW line.

C: Scatterplot for MR Analysis

This scatterplot illustrates the results of Mendelian randomization analysis between exposure and outcome factors. Each point represents an SNP, indicating 95% confidence intervals. The horizontal axis shows the effect of the SNP on exposure factors (IGFBPs), while the vertical axis shows the effect of the SNP on outcome factors (osteoporosis).

D: Forest Plot for SNP Effects

In the forest plot, each horizontal solid line represents a single SNP, estimated using the Wald ratio method. A solid line entirely to the left of 0 suggests that an increase in IGFBPx reduces the risk of developing osteoporosis, while a line to the right of 0 indicates the opposite. A solid line crossing 0 implies no statistical difference. However, it's important to note that individual SNP results may not be robust; the total IVW results are crucial.

These graphs present the results of MR studies on IGFBP1-3 and IGFBP6 as exposure factors and their relationship with osteoporosis as an outcome factor. Unfortunately, IGFBP4, IGFBP5, and IGFBP7 had too few IVs to be represented similarly.
